# Combining Co$ting Nature and Suitability Modeling to Identify High Flood Risk Areas in Need of Nature-Based Services

**DOI:** 10.3390/land10080853

**Published:** 2021-08-15

**Authors:** Sara Prybutok, Galen Newman, Kayode Atoba, Garett Sansom, Zhihan Tao

**Affiliations:** 1 Department of Landscape Architecture and Urban Planning, Texas A&M University, College Station, TX 77843, USA;; 2 Department of Marine Biology, Texas A&M University at Galveston, Galveston, TX 77554, USA;

**Keywords:** resilience, geographic information systems, green infrastructure, flood risk, spatial analysis

## Abstract

Coastal areas are often subject to the severe consequences of flooding from intense storms or hurricanes. Increases in coastal development have amplified both flooding intensity and negative impacts for coastal communities. Reductions in pervious land cover and replacement with impervious ones have reduced the amount of ecosystem services. This research examines the services provided by nature-based solutions by applying outputs from Co$ting Nature models into suitability models to quantify ecosystem services along the Texas Coast. Results show that only around 13% of the Houston-Galveston coastal area has relatively high NBS, and nearly $\frac{1}{4}$ of the area shows relatively low NBS. The majority of the areas lie in the middle, which, due to increases in development, are at particular risk for becoming areas offering low NBS in the future if not treated. Such vulnerability assessment informs future implementation strategies for NBS in coastal communities to protect people and property from flooding.

## 1. Introduction

Much of the natural coastal landscape has been rapidly converted to urbanized area over the last few decades, causing and amplifying major flood issues for cities in close proximity to the coast [1]. This is particularly problematic for fast growing coastal urban communities. For example, extremely high levels of urban development have occurred in Houston, TX, which had the largest urban growth in the US from 2001 to 2017, a pattern that has increased the probability of extreme flooding events by a factor of nearly 21 times [2]. In the same city, developed land cover increased by 21% from 1984 to 1994, 39% between 1994 and 2000, and 114% between 2000 and 2003, resulting in decreased vegetated land cover in coastal areas [3]. Increases in the vast amount of impervious surface impedes water infiltration, increases surface stormwater runoff, and causes a greater frequency and intensity of flooding [4]. Such conditions are international issues. For example, many coastal areas in the Mediterranean are suffering from flash flooding problems due to increased urbanization [5]. Further, intense development due to tourism increases in the Canary Archipelago of Spain have resulted in intense negative environmental issues and increased flooding problems [6]. National parks located in Spain’s Canary Islands are being slowly eroded by peripheral development and re-vegetation of efforts within the floodplain have been hampered by torrential floods [7]. Portugal is undergoing similar circumstances. On the 20th of February of 2010, Portugal’s island of Madeira was hit by torrential rainfall that triggered catastrophic flooding which accounted for 45 deaths; such flooding issues are projected to increase due to lack of green space, increased impervious surfaces, and forecasted climate changes [8].

Natural landscapes (such as wetlands) provide ecosystem services such as decreased stormwater runoff amounts, increased groundwater sequestration, and natural phytoremediation of contaminated water [9]. A decrease in vegetated land cover area, however, also decreases such regulating services [10]. The consequences of declines in the natural landscape quantity and quality can been seen when assessing the Houston-Galveston Metropolitan Area’s (H-G MSA) coastal cities and neighborhoods, which are also often subject to severe flooding. This presents a need for an increase in nature-based solutions (NBS) to protect these coastal cities and neighborhoods

NBS apply ideas inspired or supported by nature to the urban environment, thereby incorporating natural features and processes in solving complex environmental problems [11]. Flooding in coastal communities can result in sediment-laden water and industrial pollution mixed with stormwater runoff [9]. Such circumstances can be mitigated through the provision and protection of ecosystem services [12]. Due to high energy and resource inputs involved in traditional physical and chemical remediation methods, which can also often lead to damage of land functionality and cause secondary pollution, NBS offer a promising alternative for lowering urban flood risk and acting as resilient urban infrastructure [13,14]. Through NBS such as constructed wetlands, phytoremediation, or bioremediation, contaminated land from harmful pollutants can be treated while stormwater runoff amounts are simultaneously decreased [13].

In order to implement NBS strategies, it is important to assess the most critical areas where natural interventions are necessary. This research applies the Co$ting Nature model to the H-G MSA to identify areas that are experiencing high flood risk and low-level ecosystem services. Co$ting Nature is a web-based tool used for measuring natural capital, analyzing ecosystem services granted by nature, identifying the benefits of the services, and evaluating the effects of human intervention [15]. Due to the decrease in pervious area within the H-G MSA coastal region, and subsequently, their inherent reduction in ecosystem services, the communities and neighborhoods of this area are left more vulnerable to flooding. We assess the services produced by nature-based solutions for coastal cities and neighborhoods and quantify these services through spatial analytics. Utilizing Co$ting Nature’s spatial analyses for conservation priority, ecosystem service provisions, and relative threats, we identify vulnerable coastal areas within the H-G MSA. We then conduct a suitability model from the Co$ting Nature outputs to synthesize areas performing nature-based services and overlay this output with current flood risk.

## 2. Literature Review

### 2.1. Applications of Co$ting Nature

Co$ting Nature models prioritize the conservation of ecological spaces at local, regional, and global scales based on biological importance, realized ecosystem service provisions, current pressures, and future threats. The models analyze various ecosystem services using remote sensing datasets of phenomenological models and perform calculations in biophysical units and indexes (based on binary scores) for bundling services. It utilizes more than 150 input factors (via maps) and spatial models while creating, visualizing, and analyzing downloadable datasets of each output.

Evidence-based environmental decisions can be significantly influenced by valuating both potential and realized ecosystem services [16]. For example, Aziz and Van Cappellen (2019) [17] used the Co$ting Nature model to distinguish potential and realized ecosystem services for the Southern Ontario region. The study combined spatial distribution and use-intensity of a bundle of six ecosystem services including water provisioning and supply, water quality, carbon sequestration, carbon storage, flood regulation, and nature-based tourism. The results showed that realized ecosystem services comprise roughly half of the bundled ecosystem services’ potential value.

Choi et al. (2018) [18] applied Co$ting Nature throughout the entire country of South Korea. Co$ting Nature was used to identify existing and potential conservation areas and theory related to different ecosystem services in order to assist in decision-making about national resource management. The software helped prioritize land use planning by biological importance due to its capabilities to rapidly quantify ecosystem services and identify areas of low or high priority for ecosystem service conservation.

Hemati et al. (2020) [19] applied Co$ting Nature in the Karaj district of the Alborz Province in northwestern Iran to assist in the protection of current and future biodiversity. The authors created an integrated dynamic model to produce prioritized nature conservation maps based on ecosystem services and developed simulations of future land use/land cover change for the study area. Hemati et al. (2020) [19] then used the model to prioritize conservation areas, quantify ecosystem services, and project land use and land cover changes. Results showed that if the current land cover change pattern continues, major portions of important habitats and inherent ecosystem services will be lost.

In Liu et al.’s (2017) [20] application of Co$ting Nature, the authors comparatively quantified the various ecosystem service provisions of two different conservation management approaches in Wanglang National Nature Reserve, a protected nature reserve in China, as well as a nearby Natural Forest Protection Project. The results emphasized the significance of strictly protected nature reserves in China for their extremely valuable ecosystem services as well as highlighted the benefits of their inherent carbon storage and recreation opportunities; the ecosystem services were shown to provide both national and global benefits.

Neugarten et al. (2021) [21] used Co$ting Nature to establish a framework to ascertain areas that delivered multiple ecosystem-based benefits. The framework was applied in Madagascar due to its high levels of biodiversity and human dependency on ecosystems. The findings of the study revealed that all the key biodiversity areas in Madagascar deliver several benefits to people such as biomass carbon stock, hunting and non-timber forest products, and nature tourism; however, the various sites identified differed in significance for their services.

### 2.2. Gaps in the Literature

Because locational spatial data and the distribution of ecosystem services are often-times unclear, the full protection of ecosystems is not always captured. Due to this lack of spatially explicit data, mapping ecosystem services to advise investment conservations is crucial for guaranteeing that the co-benefits are preserved. While applications of the Co$ting Nature model are limited in the literature due to its relatively new creation, much of the current applications are simply adjustments of the model’s internal inputs to streamline a strategic output. To our knowledge, there have been no connections to other spatial analytic methods, such as suitability modeling, to create targeted overlays to solve existing built environment issues. Such an approach, when focused on flood risk modeling, should be a normalized approach to combining existing areas of ecological services, potential areas of ecological services, and areas at risk of future hazard evens (in this case, flooding). Such a framework will allow planners, researchers, and policy makers the ability to (1) identify natural areas in need of immediate conservation, (2) target communities with high flood risk and low ecological services, and (3) develop new polices for instituting green infrastructure and natural conservation approaches within these identified and targeted areas.

## 3. Methods

### 3.1. Data Collection and Study Area

**Figure 1.**
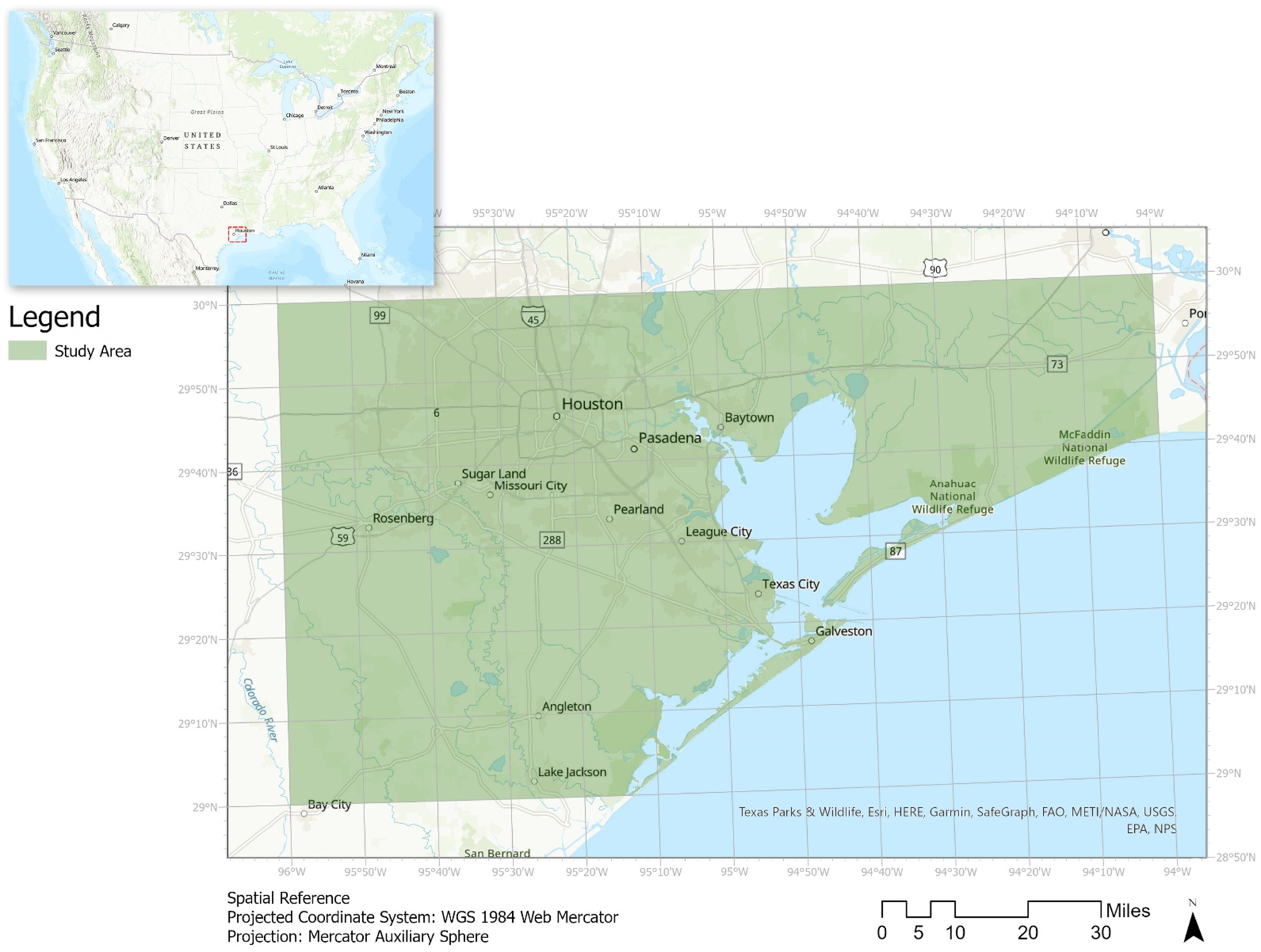
Map of the location of our study area based on Co$ting Nature’s tile extents (Figure credit: authors).

We began our spatial analysis by identifying locations where NBS can be most useful along the H-G MSA coastline (See Figure 1) by collecting data from various open geo-database sources. The primary data sources came from Policy Support System [22–26], the Co$ting Nature Tool, the Houston-Galveston Area Council (2021), the Center for Texas Beaches and Shores (2020), the National Oceanic and Atmospheric Administration (2019), and Texas Parks and Wildlife Department (n.d.) (See Table 1). These offered much downloadable data to input into ArcGIS Pro 2.7.0, primarily in the forms of raster and feature class datasets. We also accessed Policy Support System, primarily focusing on their Co$ting Nature (V3) program to value the ecosystem service and the role they played in risk management of coastal flooded, conservation, and priority areas. To encompass the entire Houston area and Galveston Bay, we selected two adjacent 1-degree tiles at 1-hectare spatial resolution from Co$ting Nature. The two tiles share a boundary line that runs from 30.0° (to the N), 29.0° (to the S). The tile that had Galveston within its boundaries used the program’s first-version mask and digital elevation models while Houston used the latest of both components for their data models. We then spatially joined these tiles together in ArcGIS Pro.

### 3.2. Data Analysis

Both tile simulations were run using the data provided by Co$ting Nature to calculate the conservation priority baseline and spatial model’s index values locally within the analysis area, although datasets slightly varied between the two tiles due to data availability. We grouped the output maps into two separate categories—eco-service and hazard mitigation maps. The eco-service map category consisted of aggregate nature conservation priority, delphic conservation priority, and bundled services output result maps; the hazard mitigation category included the relative ecosystem service (ES) relevant risk output results map. Each of these outputs, provided by Co$ting Nature, have been used in all of the previously mentioned applications of the model discussed in the literature review, to varying degrees. These are all consistent outputs provided by the model which can then be integrated, combined, or further analyzing using various spatial analytics tools.

The eco-service maps provided by Co$ting Nature are denoted as normalized biophysical units in relative terms as indices from 0–1 locally within the individual study area tiles. The aggregate nature conservation priority outputs indicate areas’ potential or actual value for services combined with nature conservation priority corresponding to perceived value and risk of loss. The bundled services outputs identify areas of total potential services as well as areas of realized services based on the greatest amount of component services in each pixel (e.g., carbon, nature-based tourism, water, etc.). The delphic conservation priority outputs represent areas of priority for conservation based on the overlap of institutional conservation priorities for major conservation non-governmental organizations (NGO). Finally, for the hazard mitigation outputs, the relative ecosystem service (ES) relevant risk map is represented in the same way as the eco-service maps; the relative ES relevant risk output map shows areas of risks towards ecosystem services.

**Figure 2.**
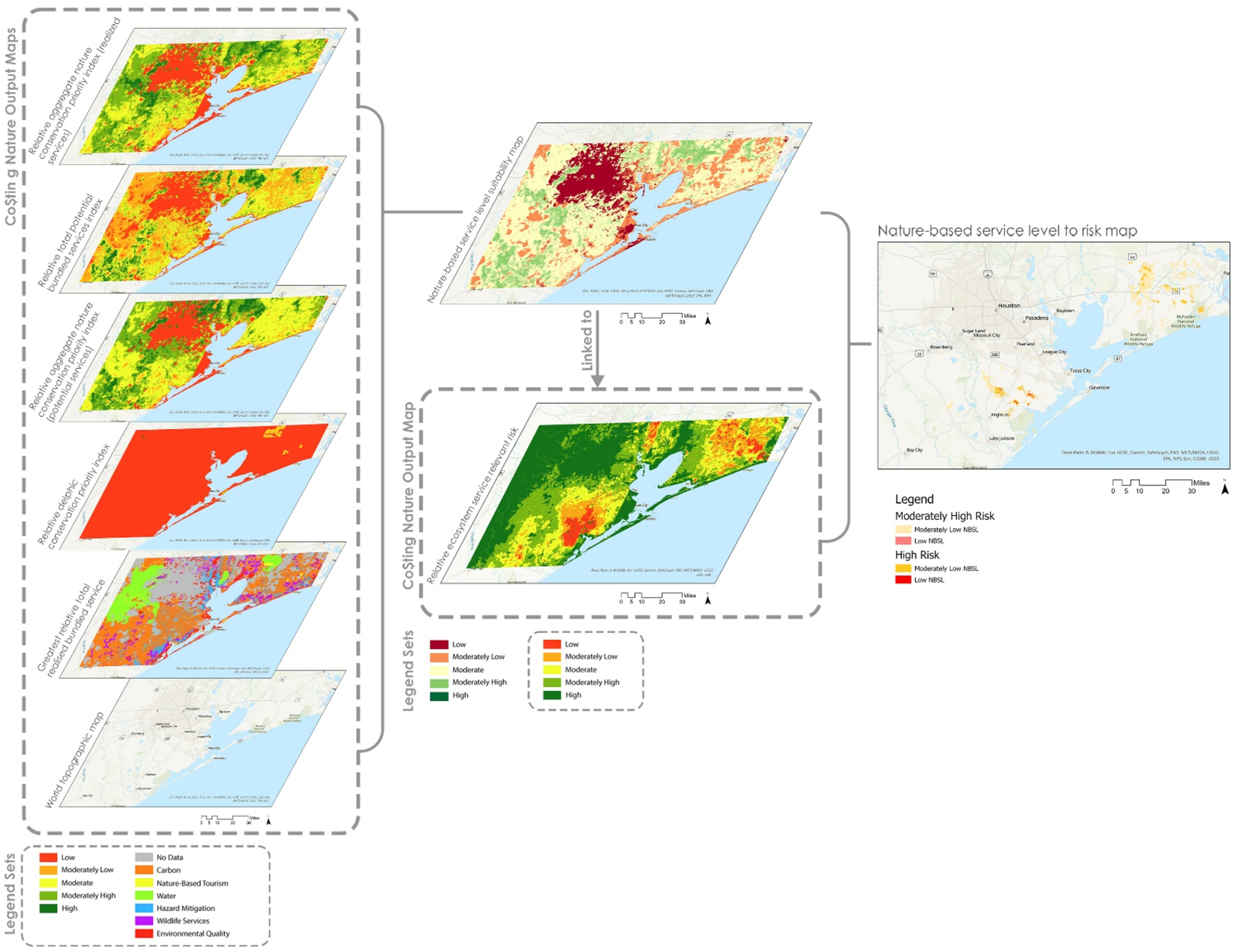
Exploded axon showing suitability model using Co$ting Nature outputs used to determine coastal areas with low nature-based solutions and high flood risk.

Since not all the services in the greatest relative total realized bundled service output, and the predominant realized service in each pixel were applicable to this study’s objectives, we extracted carbon, nature-based tourism, water, hazard mitigation, wildlife services, and environmental quality as our primary outputs for analysis. We then reclassified these eco-service maps with an index value range into five categories each. A “1” score indicated the lowest level of service provided (least suitable), while a “5” score signified the highest number of services (most suitable). The hazard mitigation map of relative ES relevant risk was also classified by a similar system where a “1” score denoted a low risk while a “5” score represented a high risk. An equally weighted overlay between all five of the Co$ting Nature eco-service output maps was then applied. Because we treated both risk and NBS as equally important, the weights were uniformly applied to create the suitability model. The equally weighted approach has been routinely applied when conducting similar spatial overlays in the literature and such an approach helps limit biases in the outputs which can come when overly weighting specific variables [27–29]. Overall, we weighted 5 of the Co$ting Nature outputs at 20% each (relative aggregate nature conservation priority index (potential services), relative total potential bundled services index, relative aggregate nature conservation priority index (realized services), relative delphic conservation priority index, and greatest relative total realized bundled service), to create a 100% weighting summation, then linked this output with the relative ecosystem services relevant risk output (See Figure 2). The numeric classification breakdown for the eco-services and hazard mitigation groups’ maps as well as their descriptions given by Co$ting Nature used to create the nature-based service level suitability model are shown in Table 2.

The output of this overlay was then integrated with the Co$ting Nature flood risk map to determine coastal areas within the H-G MSA that have low ecosystem services but high flood risk, thereby identifying neighborhoods in dire need of immediate NBS. The weighted overlay output map of the NBS levels overlaid with the relative ES relevant risk output map result can be seen in Figure 2. This risk matrix within this figure shows NBS classifications allowed, as well as the regions and areas of greatest risk (moderately high and high) against all levels of nature-based service, along with the existing NBS (low and moderately low) that overlap high flood risk areas (moderately high and high).

## 4. Results

**Figure 3.**
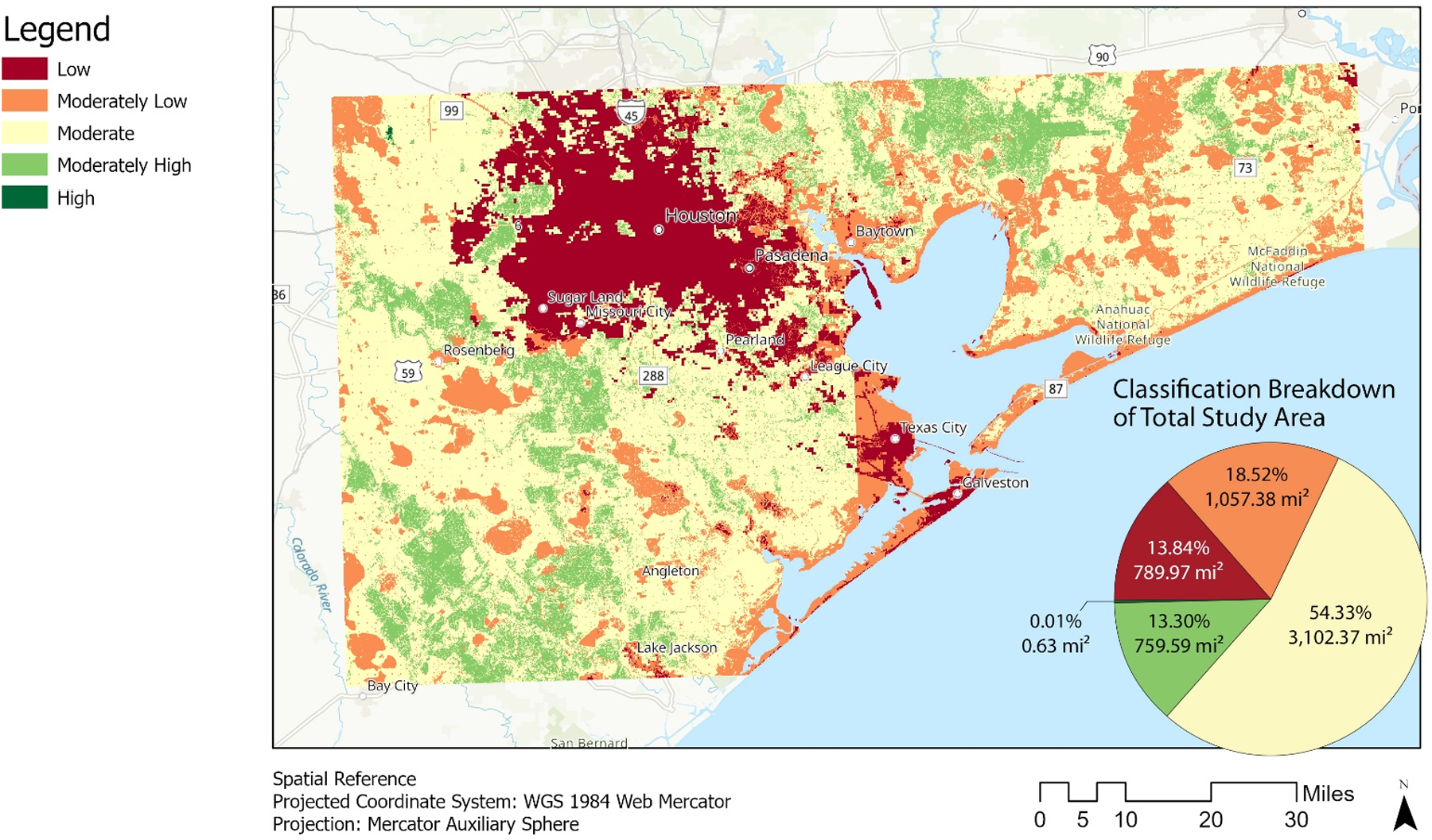
Nature-based service level outputs and their classification’s area in square miles and percent of total area of the study area derived from the number of services provided for the Houston-Galveston study area of 30° N to 29° N (north to south boundary) and 96° W to 94° W (west to east boundary).

We show the weighted overlay output of NBS levels in Figure 3 along with the class values’ percent of total area in the study area in the pie chart graphic. The outputs indicate that most areas with the lowest amount of NBS are already highly developed and populated (e.g., urbanized areas for Houston and Galveston), comprising 13.84% of the total study area. The moderately low class, with the second lowest NBS level, contains around 18.52% of the study region and forms a semi-perimeter along the H-G MSA’s coastline. At 54.33% (and nearly three times that of the second leading class area), the moderate class of NBS level has the largest area out of all the classes and encompasses much of the interior area of our study region but borders most of the moderately low-class areas. Moderately high-class areas are mostly inland and account for 13.30% of the area. Finally, the highest NBSL has the least amount of area in our study at 0.01% and is concentrated primarily only within the northwest corner of our study region. Overall, only around 13% of the H-G MSA coastal area has relatively high NBS, and nearly $\frac{1}{4}$ of the area shows relatively low NBS (See Figure 3). The majority of the area lies in the middle range, which, due to increases in development, are more than likely at a higher risk for becoming areas offering low NBS in the future, if not treated.

**Figure 4.**
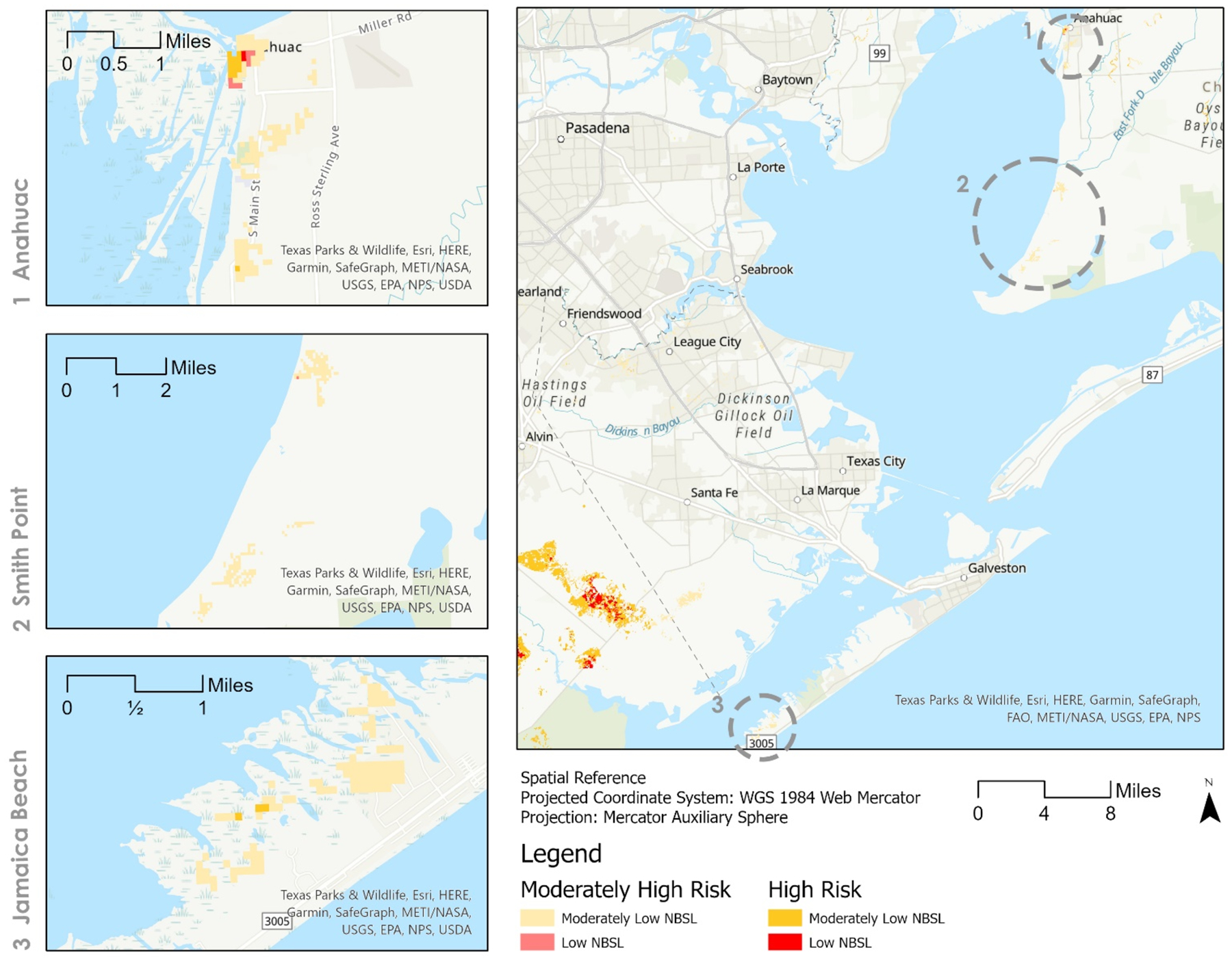
Close-ups of the three identified communities (Anahuac, Smith Point, Jamaica Beach) in relation to the whole study area that are most vulnerable to flooding due to having low to moderately low NBSLs with moderately high to high relative ES risks.

From the overlay matrix, we identified several communities that are most vulnerable to flooding due to their location on the coast, having low to moderately low NBS levels with moderately high to high disaster event risks. Based on the closest geolocation marker to the affected area, communities such as Jamaica Beach, Smith Point, and Anahuac are in dire need of nature-based provisions (See Figure 4). To detail out these conditions, the most southern site is Jamaica Beach, located on Galveston Island, which consists mainly of class 2 NBSL to class 4 risk with small areas of class 2 NBSL to class 5 risk. The furthest site north is Anahuac, with a combination of all four classes but predominantly class 2 NBSL to class 4 risk. The last site situated between the two priorly named communities is Smith Point, which is composed of class 2 NBSL to class 4 and a single area of class 1 NBSL to class 4 risk.

## 5. Discussion and Conclusions

With the impending threat of flooding and many areas within the H-G MSA having a diminishing natural landscape due to development, there is a high importance on implementing nature-based solutions along the coast to protect vulnerable neighborhoods. In the study, we analyzed nature-based service levels concerning risks based on aggregate nature conservation priority, delphic conservation priority, bundled services, and a relative ES relevant risk output results map which were provided by the Co$ting Nature model. Through applying a suitability model to the Co$ting Nature outputs, we identified high flood risk and low ecosystem service areas in need of immediate NBS to increase resilience. As an example, three vulnerable community and neighborhood locations requiring the addition of NBS were shown as example neighborhoods: Jamaica Beach, Smith Point, and Anahuac. Areas experiencing such circumstances (e.g., low levels of nature-based solutions and high hazard risk) indicate a potential need to increase nature-based solutions through policy implementation at local levels.

Local planners and decision-makers play a vital role in promoting NBS to manage and mitigate flood risk through funding, policies, and incentives. Examples include policies geared towards property acquisition and open space restoration [30], strategies that have been recommended for the study area in recent studies [31,32]. By constructing and implementing such NBS in high-risk communities where little exists currently, these neighborhoods can increase their resilience to flood events. Methods to help manage and mitigate flood risk through NBS include sustainable urban drainage systems (SUDS), low impact development (LID), water sensitive urban design (WSUD), and nature reserves [13,33] (p. 123).

In areas experiencing the aforementioned conditions, which have seen repeated damage due to flood events in the past, buyout programs which leverage federal funding to purchase flood-prone properties and restore them to vacant open spaces are a potential option for NBS in the Houston area. However, existing buyout policies are flawed in their implementation of open space restoration and exacerbate existing social inequities [34,35]. To avoid this, buyout programs can integrate NBS strategies rather than leaving a patched distributed of open spaces across coastal communities. For example, Atoba et al. (2020) [31] propose annexing existing ecosystem services such as parks, wetlands, conservation easements, and floodplains to mitigate the effect of flooding and increase ecosystem services. For example, after Hurricane Floyd hit Kinston, North Carolina, properties purchased for buyouts were to be converted into open space with a connecting nature trail, picnic areas, hiking and biking paths, and a tourist Civil War site [36]. Additionally, efforts towards conserving open spaces by purchasing already at-risk properties before development occurs [32] can also be a viable NBS strategy.

Another form of NBS-related policy can be implemented through LID to improve resilience against climate change, especially in already urbanized areas. LID is a type of stormwater management practice which retains water onsite and utilizes the natural layout of the site to enhance filtration, encourage phytoremediation, and increase stormwater retention. There are two types of LID practice—structural mechanisms (e.g., green roofs, rain gardens, constructed wetlands, native vegetation) and nonstructural (e.g., rain barrels, oversized drainage pipes, pervious paving materials) [37]. To assess LID impacts, a study on a redevelopment project in south of Boston, Massachusetts by Pyke et al. (2011) [38] simulated stormwater models using the Smart Growth Water Assessment Tool for Estimating Runoff across various precipitation scenarios. The results showed that LID sites managed stormwater runoff and pollutant from all precipitation scenarios better than conventionally developed sites [38]. LID practice is more effective in mitigating impacts of urbanization and land developments on hydrology and water quality when efficient design, implementation, and maintenance are utilized; it is also best employed in conjunction with traditional stormwater management approaches [37,39]. Some LID techniques are already being applied in the study area, with findings showing that some LIDs can reduce discharges during selected storm events when implemented at a large watershed scale [40].

This paper presents an important step in identifying flooding and development vulnerabilities in coastal communities by proposing a model that can be easily replicated by other researchers and local planners. With both Co$ting Nature and ArcGIS accessible, this model can be easily utilized and reproduced by both researchers and practitioners. While this paper provides potential opportunities for modeling NBS, additional research and data analytics can be conducted in future studies to address some of the limitations in this study and further advance this area of enquiry. For example, Co$ting Nature have a couple of limitations such as: failing to highlight the actual monetary value of the identified ecosystem services [41], problems with weighing of land use categories and ecosystem services [17,18], and constraints in input data requirements and policies for local applications [17,19]. However, despite these limitations, Co$ting Nature effectively identifies specific areas for conservation, locates both existing and potential ecosystem service provisions, and characterizes geographic extents according to their specific ecosystem services (e.g., hazard mitigation, carbon sequestration, nature-based tourism, etc.). This adequately address the need for identifying vulnerable areas for the use of NBS in coastal communities. Finally, future studies should also explore how the various configurations of NBS in conjunction with other flood mitigation strategies can perform as a synergistic approach to flood risk reduction and hazard mitigation. It should be noted that while the areas identified in this research intersect with flood risk and low ecosystem services, additional information may be needed to justify the use of future NBS. For example, engineered flood infrastructure such as levees and stream hardening to increase peak flow may prove to be more useful in some areas than NBS as future studies are conducted. While NBS are more applicable, a synergistic combination of several flood mitigation strategies needs to be employed.

## Figures and Tables

**Table 1.** Primary datasets’ dates and sources for the layers used in preliminary research and background information towards creating the suitability model for our study area.

Datasets	Date	Data Sources
NLCD Land Cover	2001–2019	Multi-Resolution Land Characteristic Consortium
Land Cover	2010–2011	The Center for Texas Beaches and Shores (CTBS)
Flood Vulnerability	2013	The Center for Texas Beaches and Shores (CTBS)
Hazards and Hazard Mitigation	2011	Co$ting Nature
Key Output Maps	2011	Co$ting Nature
Workspace Data	2011	Co$ting Nature

**Table 2.** The breakdown of numeric classification for the eco-services and hazard mitigation groups’ maps as well as the descriptions given by Co$ting Nature used for the suitability model.

Group	Map Title	Description	Weighted Overlay (%)	Numeric Classification
Eco-Services	Relative aggregate nature conservation priority index (potential services)	Pressured and threatened conservation priority areas with high potential service provision into five equal value classifications for the combined Houston and Galveston focus areas.	20	1: ≤0.20 and No Data 2: ≤0.40 3: ≤0.60 4: ≤0.80 5: ≤1.00
	Relative total potential bundled services index	Total potential services including water, carbon, nature-based tourism, hazard mitigation services, timber (commercial and domestic), fuelwood, grazing, wildlife services, non-wood forest products, wildlife dis-services, aquatic fisheries (commercial and artisanal), and environmental quality into five equal value classifications for the combined Houston and Galveston focus areas.	20	1: ≤0.20 and No Data 2: ≤0.40 3: ≤0.60 4: ≤0.80 5: ≤1.00
	Relative aggregate nature conservation priority index (realized services)	Pressured and threatened conservation priority areas with high realized service provision into five equal value classifications for the combined Houston and Galveston focus areas.	20	1: ≤0.20 and No Data 2: ≤0.40 3: ≤0.60 4: ≤0.80 5: ≤1.00
	Relative delphic conservation priority index	Conservation priority by overlap of EBAs (Birdlife), Global200 Ecoregions (WWF), Hotspots (CI), Last of the Wild (WCS, CIESIN), Important Bird Areas (Birdlife), and Key Biodiversity areas (IUCN, BI, PI, CI) into five value classifications (0.1, 0.25, 0.5, 0.75, 1.0) for the combined Houston and Galveston focus areas.	20	1: ≤0.10 and No Data 2: ≤0.25 3: ≤0.50 4: ≤0.75 5: ≤1.00
	Greatest relative total realized bundled service	Greatest realized service from: water, carbon, nature- and culture-based tourism, hazard mitigation, timber (commercial and domestic), fuelwood, grazing, wildlife services, non-wood forest products, wildlife dis-services, aquatic fisheries (commercial and artisanal), and environmental quality for the combined Houston and Galveston focus areas.	20	1: No Data 5: Carbon 5: Nature-Based Tourism 5: Water 5: Hazard Mitigation 5: Wildlife Services 5: Environmental Quality
Hazard Mitigation	Relative ecosystem services (ES) relevant risk	Relative ecosystem service (ES) relevant risk (exposure × vulnerability), 0–1 locally into five equal value classifications for the combined Houston and Galveston focus areas.	N/A	1: ≤0.20 and No Data 2: ≤0.40 3: ≤0.60 4: ≤0.80 5: ≤1.00

## Data Availability

Data for the study can be obtained from http://www.policysupport.org/costingnature (accessed on 13 March 2021) with permissions from the software and modeling company.
